# Quantifying host-microbe interactions with bacterial lineage tracing

**DOI:** 10.1126/science.adx5362

**Published:** 2026-01-01

**Authors:** Ian W. Campbell, Karthik Hullahalli, Matthew K. Waldor

**Affiliations:** 1 Brigham and Women’s Hospital Division of Infectious Diseases and Harvard Medical School Department of Microbiology, Boston, MA, USA; 2 Howard Hughes Medical Institute, Boston, MA, USA; 3 Current address, Loyola ssUniversity Chicago Department of Microbiology and Immunology, Maywood, IL, USA

## Abstract

Using genomic barcodes to trace bacterial lineages within a host reveals previously unobservable dynamics of infection, including the impact of infection bottlenecks, routes of bacterial dissemination, and patterns of within-host evolution. Barcoding introduces trackable diversity to otherwise isogenic bacterial populations. Comparing the barcodes within an inoculum to those within the host quantifies the ‘founding population’, which reveals the magnitude of population collapse caused by host bottlenecks. Furthermore, comparisons of the founders between tissues can reveal the patterns of pathogen dissemination. On longer timescales, the emergence of dominant barcoded lineages can also be used to detect within-host evolution. Collectively, barcoding studies quantify the hidden parameters that underlie bacterial colonization and creates a quantitative framework for modeling and preventing infectious disease.

## Enhanced Abstract

**Background:**

Following exposure to bacteria, individuals can become infected if some of the encountered microbes persist and replicate in the host. These ‘founders’ are the organisms that survive host defense processes, and their subsequent adaptation, replication, dissemination, and evolution govern the outcomes of infection. Despite their central importance in establishing infections, the identity of the founding organisms in a tissue is unobservable by traditional colony counting because such bulk measurements of bacterial numbers do not monitor individual clones. DNA barcoding overcomes this observational limitation by introducing genetic diversity without altering bacterial fitness. Analyses of barcode abundances determined by DNA sequencing reveal the identity of the underlying founding population and facilitate tracing the fates of the founders within and between hosts. Barcode-based approaches enable the monitoring of individual clones and the quantification of otherwise hidden facets of host-microbe interactions.

**Advances:**

DNA sequencing-based lineage tracing offers a method for analyzing the dynamics of bacterial populations within the host, providing insights into the nature of infection bottlenecks, routes of bacterial dissemination, and patterns of within-host microbial evolution. The relationship between the dose of infectious challenge and the size of the founding population revealed by barcoding studies directly quantifies infection bottlenecks, which eliminate inoculated microorganisms prior to the establishment of founding populations. By quantifying the infection bottleneck, barcoding studies have uncovered host and microbial factors that determine susceptibility to infection, including the roles of stomach acid, innate immunity, vaccination, microbiota, bacterial motility, and microbial quorum sensing. Furthermore, by comparing the identity of founders isolated from different tissues, barcoded bacteria can reveal the paths of pathogen spread and provide high-resolution maps of when and where bacteria disseminate within the host. These studies have identified host and bacterial factors that control dissemination and host compartments that efficiently exchange bacteria; for instance, the gallbladder is a common reservoir for dissemination. Over longer time scales, barcodes can reveal the emergence of dominant lineages, which indicate the evolution of bacterial genotypes within the host in response to factors, including stress, inflammation, and pressure from the microbiota. Collectively, lineage tracing studies quantify the hidden parameters that underlie bacterial colonization and infection, thereby defining new roles for host and bacterial processes in the control of host-microbe interactions.

**Outlook:**

By quantifying bottlenecks and monitoring the trajectories of individual bacterial clones, barcoding and sequencing-based lineage tracing offer a new approach to address longstanding and fundamental questions in host-microbe interactions. Such questions include understanding why some individuals are more susceptible to infection and deciphering how microbes spread within and between hosts. We anticipate that the capacity to quantify founding lineages will uncover hidden patterns underlying infection, providing a new quantitative framework for modeling and preventing disease. These insights will facilitate the development of live microbial therapeutics by providing knowledge of the host bottlenecks that impede colonization of beneficial microbes. Furthermore, barcoding studies will guide strategies to prevent disseminated bacterial infections by unveiling the paths and timing of bacterial spread. These studies will also accelerate our understanding of how within-host evolution shapes the outcome of infection, which is particularly relevant in the era of increasing antimicrobial resistance. Other promising applications of bacterial barcoding include investigating the dynamics of the microbiome, defining the impacts of microbial heterogeneity, deepening mechanistic understandings of polymicrobial infections, and using barcoded challenge strains for human studies. Additional technological advancements, such as combining barcoding with imaging or temporal control of barcode diversity, will further enhance the utility and precision of these approaches. As high-throughput sequencing technologies become increasingly accessible, we anticipate that DNA barcoding will be a routine method for studying host-microbe interactions.

## Graphical Abstract

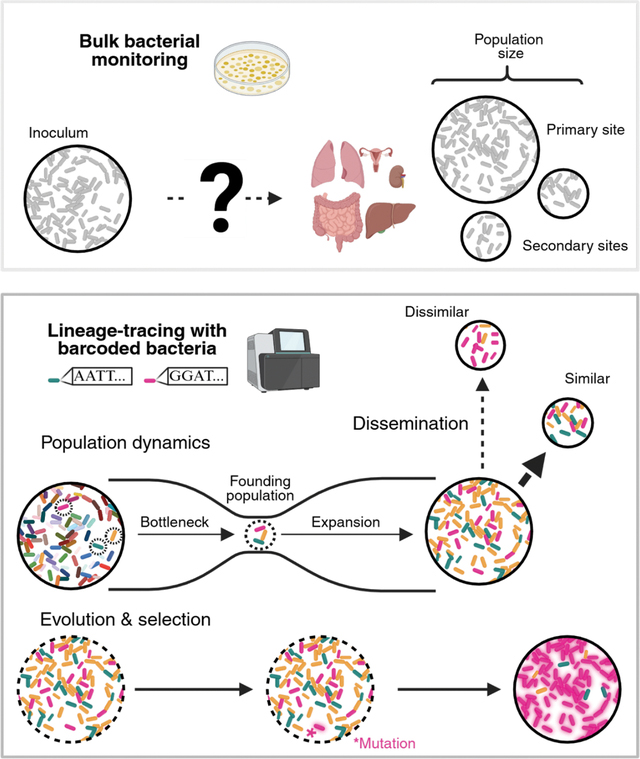

**Bacterial lineage tracing decodes infection bottlenecks, dissemination patterns, and within-host evolution.** Determining the size of the bacterial population within the host doesn’t capture the population’s history. Tracing bacteria with genomic barcodes and amplicon sequencing reveals the number of cells that initiate infection (the ‘founding population’) and quantifies bottlenecks. Comparisons of barcodes between organs reveal patterns of dissemination, and the bloom of a single lineage can reflect bacterial evolution. Created in BioRender.

For over 150 years, scientists have leveraged animal models to define how bacteria cause disease and to dissect the contributions of microbe and host (1–4). One of the key challenges for these studies is resolving the events that occur within the complex environment of an infected animal. During infection, the overlapping processes of bacterial population bottlenecks, dissemination between compartments, and evolution are challenging to disentangle with traditional bulk measurements of bacterial populations, such as counting colony-forming units (CFU), because the location and timing of these events are often unknown and are obscured by subsequent expansion of the pathogen population. Lineage tracing, defined as the monitoring of individual bacterial clones, overcomes this observational limitation.

Even before the advent of molecular cloning, researchers recognized the potential of using multiple microbial lineages to decipher the principles underlying infectivity. In 1957, Meynell and Stocker (5) conducted pioneering lineage tracing experiments using three distinct flagellar antigens of *Salmonella* to track the fate of individual clones or groups of clones. By measuring the ratio of flagellar types in a model *Salmonella* infection, the authors observed that at low doses, the relative composition of the marked lineages in tissue was significantly different from the inoculum. They proposed that these data support the theory of independent action (6–8), which conceives that each microbe possesses an independent probability to cause infection, and that the likelihood that one or more microbes initiate infection increases as the size of the inoculum increases.

In the current era of increasingly affordable high-throughput sequencing and oligonucleotide synthesis, bacterial inocula can now be subdivided into hundreds to thousands of otherwise isogenic but easily distinguishable clones using DNA barcodes, which are short DNA sequences integrated into a fitness-neutral site in the genome. Quantifying the barcodes in a population effectively enables single-cell monitoring of the clones initiating infection (9–13). The ability to monitor the fates of clones, broadly known as lineage tracing, has provided insights into mechanisms that change the likelihood of infection and expanded our understanding of the underlying dynamics of bacterial infections.

## Lineage tracing approaches in bacteria

**Fig. 1|:**
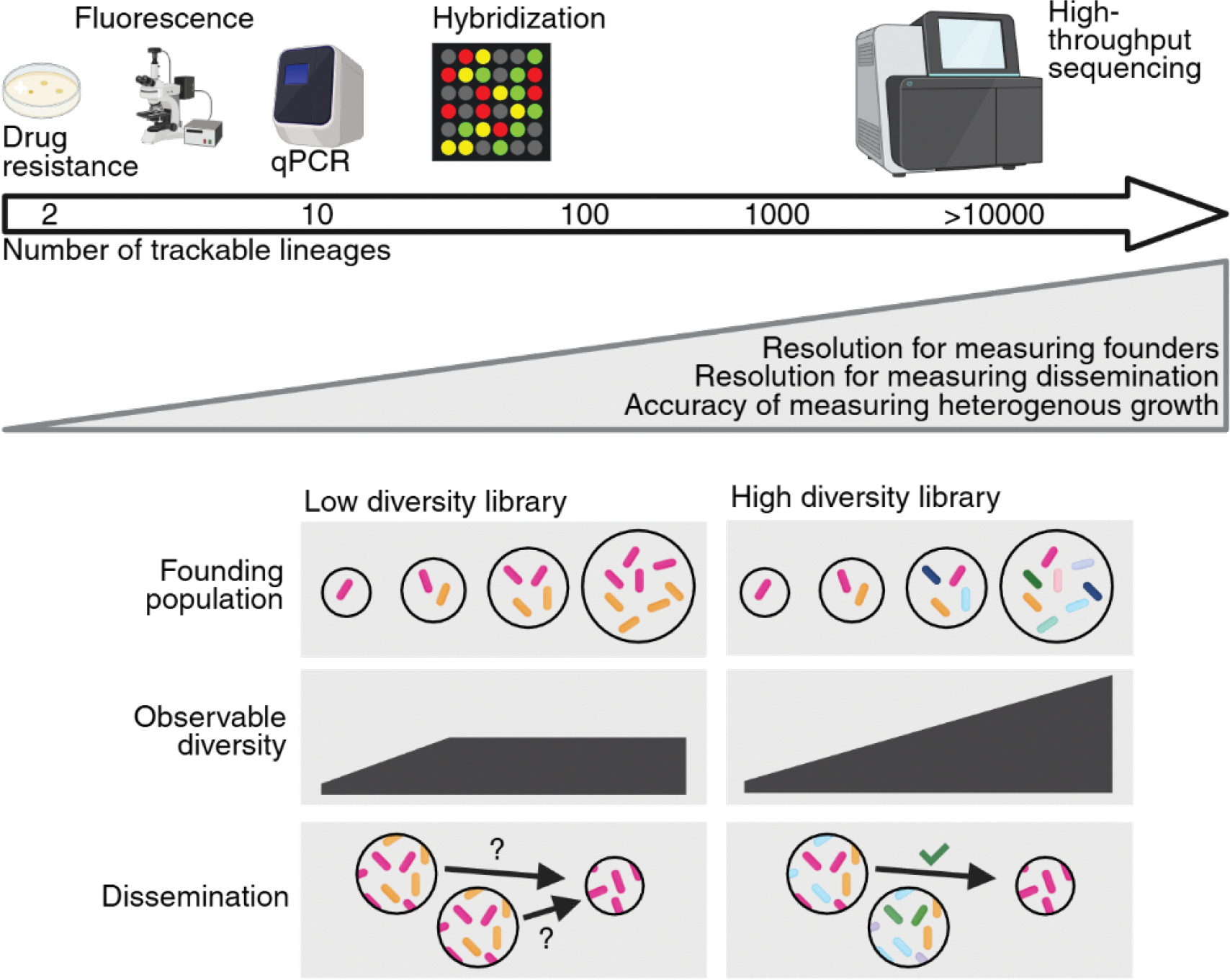
Resolution of lineage tracing calculations scales with the number of trackable lineages. The adoption of high-throughput sequencing to track genetically barcoded bacteria drastically increased the number of traceable bacterial lineages from 10s to tens of thousands, and leveraging these more diverse libraries has increased the accuracy and resolution for quantifying microbial dynamics within the host. For example, increasing the number of founders (schematized as the size of the “founding population” circle) proportionally increases observable diversity up to a higher upper bound with a more diverse library. Furthermore, a more diverse library increases the capacity to unambiguously track dissemination between organs. Created in BioRender.

The simplest lineage tracing experiments involve the use of two subpopulations. These subpopulations can be distinguished (i.e., “traced”) by several approaches, including the use of different antibiotic resistance markers, the activity of enzymes such as *lacZ*, unique fluorescent proteins, or distinct amplifiable DNA sequences (i.e., “barcodes” or “tags”; Fig. 1). Bacteria within these subpopulations are identical except at the genetic locus containing the unique sequences. Tracing lineages from the inoculum to infected tissue using these genetic markers enables the estimation of the number of clones that initiate infection, known as the founding population (13). For example, if a host is inoculated with a 1:1 mixture of two differentially tagged strains, but only one of the strains is consistently recovered from host tissues, it is likely that only one cell successfully initiated infection (i.e., one founder). Smaller founding populations relative to the inoculated dose indicate tighter bottlenecks – the processes that eliminate bacterial lineages from the inoculum (Box 1). Notably, here ‘founders’ are the number of unique cells that initiate infection, a number calculated from the change in the frequency and number of barcodes between the inoculum and the tissue; in contrast, ‘founders’ have previously referred to the number of unique genotypes that initiate infection, including in HIV infection (14), which could be represented in multiple cells/virions.

The number of different traceable markers (alleles) in a population is known as the diversity (often referred to as “richness” in ecology). Low-diversity populations are typically simpler and more cost-effective to monitor than high-diversity populations; for example, it is simple to distinguish a green and red fluorescent bacterial population, but it becomes very challenging to measure thousands of different colors. qPCR approaches have been applied to libraries consisting of ~8 lineages, and hybridization-based approaches have been used to quantify ~30 lineages (9, 15–17). Nevertheless, early studies using low diversity populations revealed fundamental aspects of infection dynamics in animal models of *Salmonella* and *Yersinia* infection and demonstrated the power of using barcoded bacteria to study host-microbe interactions.

While substantial insights have been gained from experiments with low-diversity differentially-marked bacteria, higher diversity libraries are advantageous because they improve resolution in determining the size of the founding population and the paths of dissemination (Fig. 1). For example, with less diverse libraries (i.e., with fewer barcodes), when the bottleneck is wider, the size of the founding population becomes unmeasurable as the bottleneck fails to change the composition of the population compared to the inoculum. Furthermore, it is more likely that bacterial populations within two distinct tissues of an animal may share the same tags by chance when using less diverse libraries, obscuring whether there is dissemination between the sites (Fig. 1). However, differentiating tags in high-diversity libraries is not practical with qPCR or hybridization-based detection and instead requires high-throughput sequencing. While increasing the power to differentiate individual lineages, the downside of high-diversity libraries is that they are generated and maintained as a pool of strains, and thus, it is impractical to individually characterize each lineage. In rare cases, even though the bacterial population was initially isogenic, background mutations can occur in the barcoding process, resulting in reproducible phenotypic differences between lineages, such as the dropout of barcoded strains with a colonization defect or the bloom of mutants with an advantage (11). Furthermore, since the abundance of each barcode is lower in libraries with more barcodes, analysis must account for interference introduced by technical artifacts (e.g., sequencing noise and index hopping).

**Fig. 2|:**
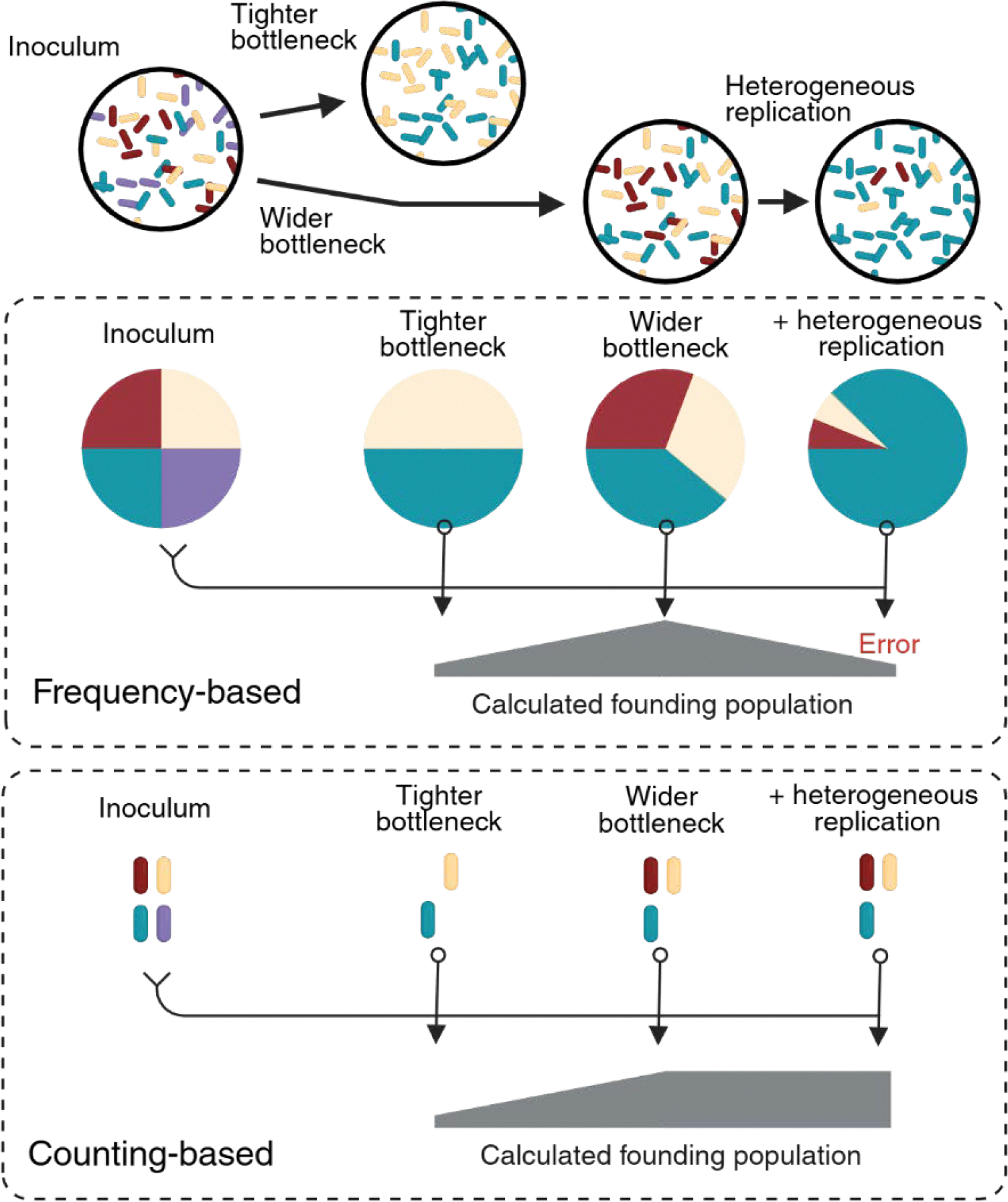
Frequency-based and counting-based methodologies for quantifying the size of the founding population. Methodologies to calculate the size of the microbial founding population from the sequencing-based enumeration of barcoded bacteria. Frequency-based approaches compare the change in lineage frequency from the inoculum to observed populations using equations from population genetics that assume greater genetic drift (i.e., greater change in lineage frequency) equates to a more stringent bottleneck and fewer founders. However, the frequency of lineages can also be impacted by heterogeneous replication, resulting in an error that underestimates the number of founders. Counting-based approaches compare the number of barcodes in an observed population (observed diversity) to simulations of the inoculum across multiple sampling sizes to determine the size of the founding population. Because counting-based approaches ignore the frequency of tags and consider only their presence or absence, they are not susceptible to errors from heterogeneous replication but require more unique lineages to achieve the same resolution as frequency-based approaches. Created in BioRender.

Several approaches have been developed to calculate the size of the founding population from read counts of barcoded bacteria; these approaches can be broadly characterized as either relying on the change in the frequency (frequency-based) or the absolute number (counting-based) of identifiable lineages (Fig. 2). For example, Sequence Tag-Based Analysis of Microbial Populations (STAMP) uses a classical population genetics analysis framework to estimate the size of the founding population from the change in frequency of tags. This frequency-based approach derives from the principle that more stringent bottlenecks lead to greater changes in the frequency of tags (13, 18). However, approaches that rely on the change in frequency of individual tags broadly assume that the only factor that contributes to variation in lineage frequency is the bottleneck and can underestimate the size of the founding population when processes other than bottlenecks change the frequency of observed lineages (Fig. 2). For example, if 10 lineages found a population, but one lineage expands to become disproportionately abundant, frequency-based calculations of the founding population will yield values less than 10, which is not an accurate measure of the size of the founding population since the detection of 10 tags indicates the existence of at least 10 founders. STAMPR (which refers to STAMP with the addition of Resampling-based calculations) refined the founding population calculation by integrating the frequency-based STAMP method with a counting-based estimate that measures the change in the number of observable lineages relative to the inoculum (Fig. 2), circumventing the errors introduced by heterogeneous replication (13, 19). To derive a counting-based estimate of the size of the founding population, STAMPR simulates bottlenecks *in silico* to determine how deeply the inoculum must be resampled to observe the number of lineages detected in tissue samples.

A notable limitation to counting-based approaches for calculating the size of the founding population is that they require a greater number of lineages to achieve the same resolution as frequency-based approaches; however, modern molecular biology enables the facile creation of tens of thousands of lineages, expanding the practical utility of counting approaches for calculating founding populations.

Generally, the upper limit to the measurable founding population is ~20 times the number of barcodes in the library when using counting-based approaches, although this number varies depending on the frequency distribution of barcodes in the inoculum. As the calculable upper limit is determined by the number of distinguishable lineages in the inoculum when using counting-based approaches, using a more diverse library will increase the resolution of lineage tracing applications. If technical limitations prohibit the creation of highly diverse libraries (e.g., low conjugation efficiencies), then experimental designs should be adjusted to account for smaller numbers of barcodes (e.g., reducing the dose or selecting a different time point).

In addition to these strategies for calculating the size of the founding population, barcodes can be used to estimate the rates of within-host bacterial replication, death, and migration, as has been modeled for invasive *Salmonella* infection in mice (12, 17). To estimate these additional parameters, these strategies require spatial and temporal measurements of barcodes along with infection system-specific knowledge, such as the evenness of replication and migration over the course of infection. When such parameters are understood for a specific infection context, these more sophisticated modeling approaches yield a highly granular assessment of bacterial infection dynamics.

## Bottlenecks and dose

Infectivity is classically determined by experimentally establishing which doses consistently cause infection (e.g., median infectious dose), with more infectious pathogens causing more infections at lower doses. Recent barcoding studies offer a new perspective on measuring infectivity (25–27). At least one founder is required for successful infection; thus, barcoding provides an experimental framework to understand infectivity by providing a method to measure the size of the founding population.

**Fig. 3|:**
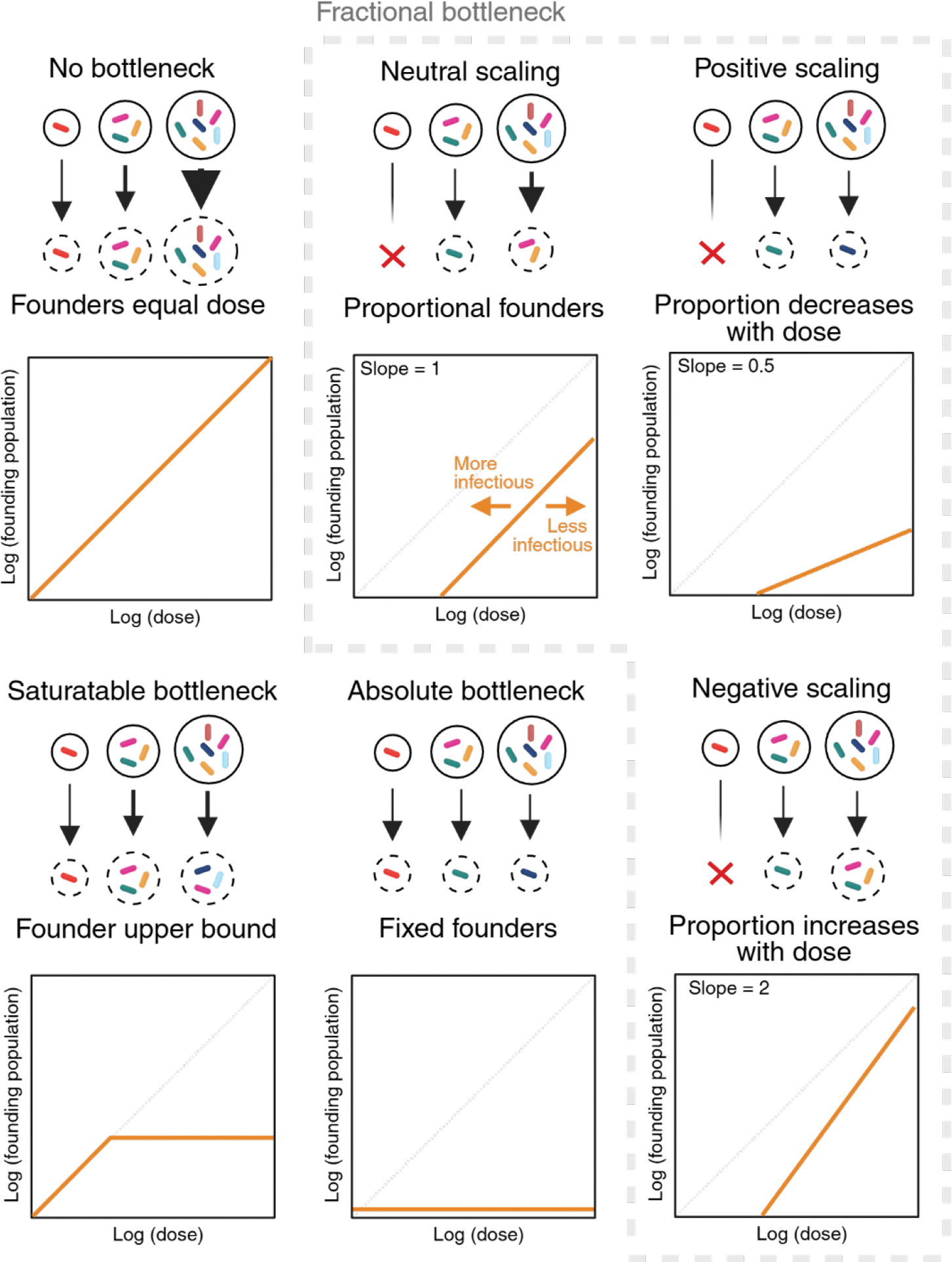
Infection bottlenecks are quantified by the relationship between dose and founding population. Bottlenecks determine the relationship between the number of bacteria that encounter the host (dose) and the number of microbial founders that initiate infection. With no bottleneck, the number of bacteria in the inoculum and the number of bacterial founders are equal at all doses. Fractional bottlenecks can be categorized as having neutral, positive, or negative scaling. With neutral scaling, the number of founders is a fixed portion of the inoculum at all doses (here depicted as one founder for every three bacteria in the inoculum). With positive scaling the proportion of founders compared to the inoculum decreases at higher doses (fractionally tighter bottlenecks), and vice versa with negative scaling. The median infectious dose is an emergent property of the curve relating dose to the size of the founding population; the x-intercept, representing the dose that yields 1 founder, is approximately equal to the dose where 50% of animals are expected to be infected with one or more founders (infectious dose 50; ID50). A saturable bottleneck has a fixed upper bound on the number of founders above which increasing the dose does not increase the size of the founding population. An absolute bottleneck restricts the inoculum down to the same fixed number of founders regardless of dose. Expanded from concepts in Abel et. al., 2015 (10). Created in BioRender.

During infection, if the bacterial population does not experience a bottleneck contraction, then the initial population (i.e., the inoculum) is equal to the size of the founding population (Fig. 3). Bottlenecks contract the inoculum down to the founding population. The relationship between dose and the founding population defines the severity of the bottleneck, with more restrictive bottlenecks eliminating more bacteria and producing fewer founders at each dose. Thus, bottlenecks can be quantified as the relationship between the founders and the dose. In most experimentally measured bottlenecks, the founding population is proportional to the dose, a relationship referred to as “fractional” (Fig. 3), since a fixed fraction of organisms in the inoculum, rather than a fixed number, establishes infection (10, 25–27). For example, the bottleneck impeding infection in C57BL/6J mice with the enteric pathogen *Citrobacter rodentium* is extremely stringent, and only ~1 in 10^8^ organisms from the inoculum survive to establish the infection and ultimately proliferate to 10^10^ cells across the intestine (25). Doses less than 10^8^ do not lead to reproducible infection because there are not enough bacteria to consistently overcome the bottleneck (~1/10^8^-fold), often resulting in 0 founders. However, based on the ~1/10^8^-fold bottleneck, it is predicted that 1 in 10 animals challenged with 10^7^ *C. rodentium* will become infected.

Fractional bottlenecks can be visualized as a curve relating dose and founding population, and this mathematical framework illuminates the underlying link between bottlenecks and infectivity (Fig. 3). Changes to the relationship between dose and founding population reflect alterations in infectivity and lead to a change in the median infectious dose, which is reflected by the x-intercept on the dose vs founding population curve. For example, *C. rodentium* is more infectious for mice treated with agents that reduce acid production in the stomach, producing ~10-fold more founders at every dose and decreasing the median infectious dose ~10-fold (25). Thus, the median infectious dose, which is classically used to define pathogen infectivity, is an emergent property of bottlenecks.

The rate at which the founding population increases as a function of dose (i.e., the slope of the dose-founding population curve) measures the efficacy of the barriers that impose the bottleneck. These curves thereby provide insight into the mechanisms of host resistance to infection. A dose-founding population curve with a slope of 1 suggests that host barriers scale in a one-to-one fashion with the size of the inoculum, referred to as “neutral scaling” (26). For example, if a neutrally scaled bottleneck constricts an inoculum of 10,000 cells into 10 founders, then raising the inoculum 10-fold to 100,000 cells will yield 100 founders. In this example, 99.9% of the inoculum is eliminated at both doses, but the number of bacteria eliminated increases from 9,990 to 99,900. Importantly, the fraction of cells eliminated is not necessarily constant at all doses. For example, it is possible that bottlenecks may tighten at higher doses (“positive scaling”) or widen at higher doses (“negative scaling”; Fig. 3) (26). These non-neutral scaling patterns reflect the mechanisms by which host bottlenecks increase or decrease their effectiveness as the challenge dose changes. One example of positive scaling is in systemic *Escherichia coli* infection, where mechanisms related to the lipopolysaccharide receptor TLR4 create a more effective barrier to infection at higher doses (26). We anticipate that further interrogation of dose-founding population curves in a variety of infection contexts will uncover additional patterns of scaling, which will reflect the diverse mechanisms by which host barriers respond to the inoculum.

In experimental models where the relationship between dose and founding population has been experimentally defined, bottlenecks have been observed to be fractional until an upper bound, defined as “saturation”, which has been hypothesized to occur when all potential founder niches are occupied (25–27). In principle, saturation could be reached at all doses above the median infectious dose, resulting in a bottleneck where the founding population is not influenced by the dose. This type of bottleneck has been termed “absolute” (Fig. 3), since a fixed (absolute) number of organisms always establishes infection. However, absolute bottlenecks in host-microbe contexts have yet to be identified with barcodes. Notably, there is an upper bound of detectable founders, determined by library size and the analytic approaches used to calculate the founding population (the ‘resolution limit’), but this is distinct from biological saturation. The fact that most experimental bottlenecks are fractional suggests that, even though distinct host factors impose bottlenecks, the quantitative properties of these factors converge towards a similar pattern.

A key advantage of lineage-tracing is that it allows differentiation of bacterial population bottlenecks from subsequent replication. This distinction can provide valuable insights into mechanisms of host defense that have important clinical implications. For example, barcoding-based studies of *Vibrio cholerae* vaccination in mice showed that a vaccination-dependent decrease in pathogen burden resulted from a tighter bottleneck that reduced the size of the founding population (28). These observations revealed that vaccination against *V. cholerae* protects by reducing the infectivity of the pathogen, which increases the median dose that can cause infection. Determining whether vaccination constricts bottlenecks or suppresses pathogen replication has critical implications for understanding vaccine efficacy. For example, if a vaccine tightens bottlenecks without suppressing replication, then infection can still occur when individuals are exposed to a sufficiently high dose. Thus, the ability of lineage tracing to distinguish the biological variables that impact bottlenecks, in contrast to replication, provides a framework for understanding how vaccination, therapeutic interventions, and host processes including trained and nutritional immunity govern susceptibility to infection.

## Host mechanisms of bottlenecks

Lineage tracing provides a quantitative approach for dissecting the underlying biology that creates bottlenecks. Here, we discuss recent applications of barcoded bacteria to understand how the host contributes to the infection bottleneck.

Barcoding studies have uncovered that the major barriers to intestinal colonization are stomach acid and the microbiota, and the relative contribution of these two factors can vary depending on the organism. For example, stomach acid imposes a tight bottleneck on *Pseudomonas aeruginosa* but has a relatively minor role in restricting intestinal colonization of *C. rodentium* and *S. enterica* serovar Typhimurium (17, 25, 29, 30). These differences could be explained by the difference in acid sensitivities between these microbes. Following transit through the stomach, enteric microbes must compete with members of the commensal microbiota; for *C. rodentium* and *S.* Typhimurium, depleting the microbiota before inoculation increases the size of the founding population at least 1,000-fold, indicating that the microbiota constitutes a major bottleneck (25, 31). The spatially segregated nature of the stomach and microbiota bottlenecks results in a sequential set of barriers that invading microbes must overcome. The sequential nature and variable magnitudes of these bottlenecks underlie how the intestinal tract protects from infection, particularly in natural infections where the dose can vary substantially. Thus, at relatively low doses, stomach acidity may significantly impact the likelihood of infection; however, at higher doses, when a greater number of microbes breach the gastric barrier, the microbiota may become more important for preventing infection.

Lineage tracing has also been used to interrogate the roles of innate and adaptive immune factors in creating microbial bottlenecks. Across multiple models of infection, depletion of immune cell subsets, genetic ablation of innate immune sensors, and depletion of specific cytokines increase the size of the founding population (24, 26, 32–39). The emerging theme from these studies is that innate immune mechanisms contribute to the control of both bottleneck stringency and total bacterial burden. A consequence of widening the bottleneck can be increased diversity of the microbes initiating infection. For example, increased pathogen diversity has been observed in mice lacking the lipopolysaccharide receptor TLR4, tumor necrosis factor (TNF) signaling, and a key enzyme in the generation of reactive oxygen species by phagocytes (CYBB) (17, 26, 37, 40). Mice lacking Rag1, which lack B and T cells, also fail to effectively bottleneck orally inoculated *L. monocytogenes*, enabling the pathogen to invade the central nervous system and cause lethal cerebral listeriosis (24). The immune system’s control of microbial diversity (i.e., by creating bottlenecks) has important evolutionary implications because diversity enables the emergence of clones with new properties, such as antibiotic resistance or heightened virulence (41).

## Pathogen manipulation of bottlenecks

While bottlenecks are generally imposed on microbes by the host, lineage-tracing has also revealed that microbial properties themselves can modulate the bottleneck experienced by the bacterial population.

Research has identified numerous bacterial factors required for virulence and colonization, but the extent to which these bacterial colonization factors thwart host bottlenecks is not well understood. Lineage tracing provides a framework to distinguish between the pathogen factors that control the establishment of founders and those that control expansion. For example, in a model of *Klebsiella pneumoniae* pneumonia, where bacteria escape the lung and disseminate to other tissues, it was found that bacteria with defective protein export into the periplasm, due to the deletion of *tatC*, also have approximately a 100-fold reduced burden in the spleen and lungs. Lineage tracing refined this observation by revealing that in the lung, removal of *tatC* both reduced replication and tightened the bottleneck ~10-fold. By contrast, in the spleen, the ~100-fold decrease in the burden of bacteria lacking *tatC* was caused exclusively by decreased bacterial expansion (40). These observations suggest that the bacterial factors that antagonize bottlenecks can sometimes be partially distinct from those required for bacterial growth.

Barcoding studies have also unexpectedly identified bacterial factors that reduce the diversity of the pathogen population within the host. For example, increasing *V. cholerae* motility by deleting *motV* relaxed the bottleneck to infant mouse colonization ~100-fold by allowing bacteria to access a larger number of niches within the small intestine, especially within the proximal crypts (42). Likewise, in a model of *Streptococcus pneumoniae* colonization in the upper respiratory tract, where barcode diversity markedly decreases over a few weeks of infection, tightening of the bottleneck is partly driven by the *S. pneumoniae blp* locus, which encodes a bacteriocin and associated immunity factors (43). The authors propose that tight bottlenecks are driven by *blp* activation in a subpopulation of cells, which ultimately kills bacteria that have yet to activate *blp* immunity and drives a decrease in barcode diversity. *BLP*-mediated killing of bacterial cells also drives pneumolysin release from killed bacteria, resulting in increased inflammation, and shows how microbial control of population heterogeneity can have meaningful consequences for infection outcomes (44). Thus, the *blp* locus in *S. pneumoniae* and *motV* in *V. cholerae* decrease bacterial diversity within the respective model systems.

## Dynamics of complex bottlenecks

Although bottlenecks are often simplified as single-step events, they can operate over longer time scales. For example, in *E. coli* systemic infection, the size of the founding population decreases over several days in most tissues, reflecting the time required for the host to clear systemic bacteria (34). Likewise, in the hours following oral inoculation of mice with *C. rodentium*, a large and diverse population is detected in the intestine. However, the diversity of barcodes rapidly and markedly decreases and ultimately stabilizes 2 days post inoculation, indicating that most cells detected in the hours following inoculation are transient and fail to establish a niche in the host (25). In contrast, an increase in founders over time can indicate that new clones disseminated to the sampled tissue from a more diverse population located elsewhere within the host; for example, the size of the *V. cholerae* founding population in the proximal small intestine in infant rabbits and infant mice increases over time, likely as a result of retrograde pathogen movement from the distal small intestine (13, 27). Likewise, in mice infected with *Listeria monocytogenes*, there is a continual increase in bacterial diversity in the brain caused by persistent invasion across the blood-brain barrier (24).

Components of bottlenecks can be broadly subdivided into those that are constant and those that respond to the inoculated bacteria (inducible). For example, the low pH of the stomach, which can eliminate microorganisms before they reach the intestine, remains largely constant during microbial transit. The constant nature of the stomach acid bottleneck contrasts with the inducible nature of the cellular and protein components of the innate immune response. For example, the processes controlled by TLR4 and TNF signaling are highly inducible by microbes during the early stages of infection, and it has been observed that these pathways reduce bacterial diversity in models of *Yersinia*, *Listeria*, and *E. coli* systemic infection (36, 37, 45). The efficiency with which these innate immune signaling pathways impose bottlenecks is intrinsically linked to the extent to which these pathways are activated in response to bacteria. Constant bottlenecks may represent processes with the capacity to eliminate bacteria in a manner that is not deleterious to the host (e.g., stomach acid, physical barriers). In contrast, inducible bottlenecks may represent processes that can potentially damage host tissues without tight regulation (e.g., inflammation). More broadly, defining the extent to which bottlenecks are inducible will provide important insights into which host processes are prioritized across different quantitative levels of pathogen stimuli. For example, in frequent natural encounters with low doses of pathogens, inducible bottlenecks may not be adequately engaged, and constant bottlenecks may be more important for conferring protection from infection.

## Modes of dissemination

Barcode-based lineage tracing studies enable high-resolution mapping of bacterial dissemination patterns during infection. Such studies have identified unexpected routes and directions of bacterial spread, common reservoirs for disseminating bacteria, and host immune factors that control dissemination (12, 13, 27, 31, 35, 36, 46). Dissemination is quantified by comparing the identity and frequency of barcodes between tissues using a genetic similarity metric, where greater genetic similarity (more sharing of barcodes) suggests increased dissemination of clones. However, evidence of shared clones is insufficient to determine the direction of spread unless these observations are contextualized by anatomy and the temporal course of infection.

**Fig. 4|:**
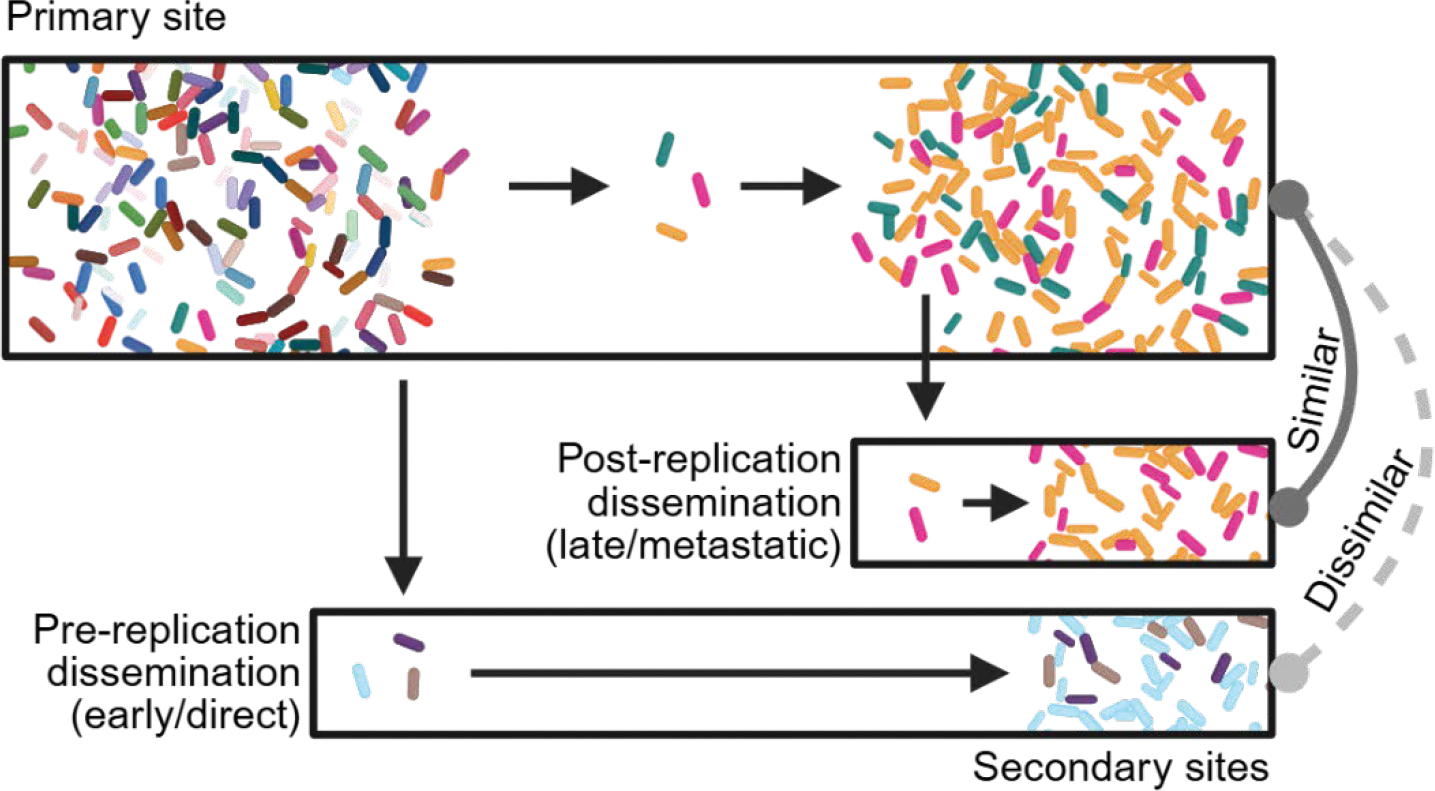
Lineage tracing measures the extent to which dissemination was preceded by replication at the primary site of infection. If dissemination occurs without subsequent replication at the primary site of infection (early or direct dissemination), then it is possible to find barcodes at the secondary site that are not present at the primary site, and consequently, the populations at the primary and secondary sites will be dissimilar. Conversely, if dissemination occurs after replication at the primary site of infection (late or metastatic dissemination), then the population at the secondary site will be similar to the population at the primary site. Created in BioRender.

The observation that the same barcode is present at two sites, assuming this is not due to chance, necessitates that the bacterial clone replicated in the primary (upstream) site prior to disseminating to the secondary (downstream) site. If a clone did not replicate at the upstream site prior to transit to secondary sites, the corresponding barcode will not be represented in the primary site, and the population at the primary and secondary sites will be dissimilar. Therefore, the mode of dissemination can depend on the extent of replication in an upstream site (Fig. 4). Replication-dependent spread, also referred to as “metastatic” or “late” dissemination, occurs following replication at an upstream site (31, 40). For example, at late time points during experimental *P. aeruginosa*, *L. monocytogenes*, and *S. enterica* serovar Typhimurium infection, pathogen dissemination to the gastrointestinal tract is preceded by replication in the gallbladder, detectable by the presence of gallbladder clones in the gastrointestinal tract (31, 36, 46, 47). In contrast, spread can also occur in the absence of substantial replication at an upstream site, termed “direct” or “early”. For example, following intravenous inoculation, *E. coli* directly translocates from the blood to the liver without replication in the blood (34). Likewise, orally inoculated *S.* Typhimurium spreads to systemic sites without substantial replication in the intestine (31). Importantly, these modes of dissemination are not a strict dichotomy, as dissemination can occur before, during, and after replication at the site of initial exposure.

The extent to which bacterial replication continues at the primary site throughout the course of infection can substantially impact dissemination patterns. For example, in a model of *K. pneumoniae* infection of the lung, disproportionately expanded lung clones more commonly escape, leading to a greater burden of translocated clones at systemic sites (40). Such “metastatic” dissemination from the lung requires the host factor Nox2, which encodes NADPH oxidase; in mice lacking Nox2, bacteria in the lung expand evenly, and lung and spleen populations are dissimilar (40). In addition, in an orogastric model of *Yersinia pseudotuberculosis,* the microbial populations in the mesenteric lymph nodes and systemic sites become more genetically similar in mice lacking TNF signaling, reflecting more translocation from the replicating populations in the intestine (37).

Barcoding studies have also revealed that the gallbladder is a common reservoir for bacterial dissemination. Notably, replication of *L. monocytogenes, P. aeruginosa*, and *S. enterica* serovar Typhimurium in the gallbladder seeds intestinal colonization and fecal shedding through the bile (31, 36, 46). Furthermore, replication in the bile/gallbladder also heightens dissemination to systemic tissues during *E. coli* bloodstream infection (34). The observation that replication in bile heightens bacterial burden at other sites underscores the importance of tracing bacterial dissemination within the host. The ability to decipher hidden paths and reservoirs of pathogen spread will enable future investigations into the mechanisms of bacterial dissemination and may lead to mitigation strategies.

## Barcoding to monitor within-host evolution

During longer-term infections, host-imposed stressors can select for the emergence of bacterial clones with mutations conferring fitness advantages. These clones can be of clinical importance, as they may represent organisms with heightened antibiotic resistance or virulence (48–52). The emergence of such clones is often closely linked with the immune status of the host, since more stringent bottlenecks in immunocompetent hosts can prevent the emergence of new lineages (53). In experimental systems, whole-genome sequencing of bacteria recovered from the host can detect the emergence of mutant clones. However, whole-genome sequencing is not practical for larger-scale temporal and spatial analysis of evolution because sampling and sequencing multiple clones is relatively expensive and laborious. In contrast, lineage tracing with barcodes, which uses amplicon sequencing, offers a simple and rapid method to determine when and where new lineages arise. Once a specific clone (barcode) becomes dominant, it can be easily isolated and sequenced to identify the causative mutation. Importantly, once evolution has decreased the diversity of the population, it is not possible to observe subsequent evolutionary events with current barcoding technology.

Barcoding has been leveraged to study how stresses drive within-host evolution in murine models of intestinal colonization. Monitoring barcoded lab-adapted *E. coli* during enteric colonization of germ-free mice revealed the rapid emergence of mutations impacting motility (e.g., deletion of *flhE-flhD*), metabolism (e.g., a frameshift mutation in *lacI*), and stress response genes (e.g., inactivation of *lon* protease). Furthermore, antibiotic administration bottlenecked the *in vivo E. coli* population, decreasing barcode diversity and selecting for antibiotic-resistant clones, which were subsequently transmitted to untreated co-housed animals (54). Likewise, barcoding has revealed that antibiotic treatment can select for bacterial mutants that enter niches less accessible to antibiotics, for instance, by increased invasion of host cells (55).

The altered selective pressures present in infected tissues can also drive the evolution of the commensal population and vice versa. In a gnotobiotic mouse model, barcoding commensal *Bacteroides thetaiotaomicron* facilitated the discovery that inflammatory disruption of the gut environment by the enteric pathogen *C. rodentium* caused the emergence of *B. thetaiotaomicron* clones with mutations that increase resistance to oxidative stress (56). By contrast, infection of mice lacking a microbiota with barcoded *C. rodentium* caused clones lacking the pathogen’s major virulence island to sweep the population, and showed that the selective pressure of the microbiota maintains pathogen virulence (25).

## Outlook

Since the advent of molecular cloning in the early 1970s, the major focus of research in microbial pathogenesis has been devoted to identifying genes that mediate and regulate bacterial virulence (3, 57). Barcoding and lineage tracing open a new perspective by enabling the enumeration of the bacteria that initiate infection and the observation of these founders’ fates. With the increased availability and reduction in the costs of high-throughput sequencing and oligonucleotide synthesis, we anticipate that existing barcoding technology will be leveraged for new applications and that new methods of lineage tracing will be developed, collectively expanding the range of questions and the power of studies addressing microbial population dynamics.

**Fig. 5|:**
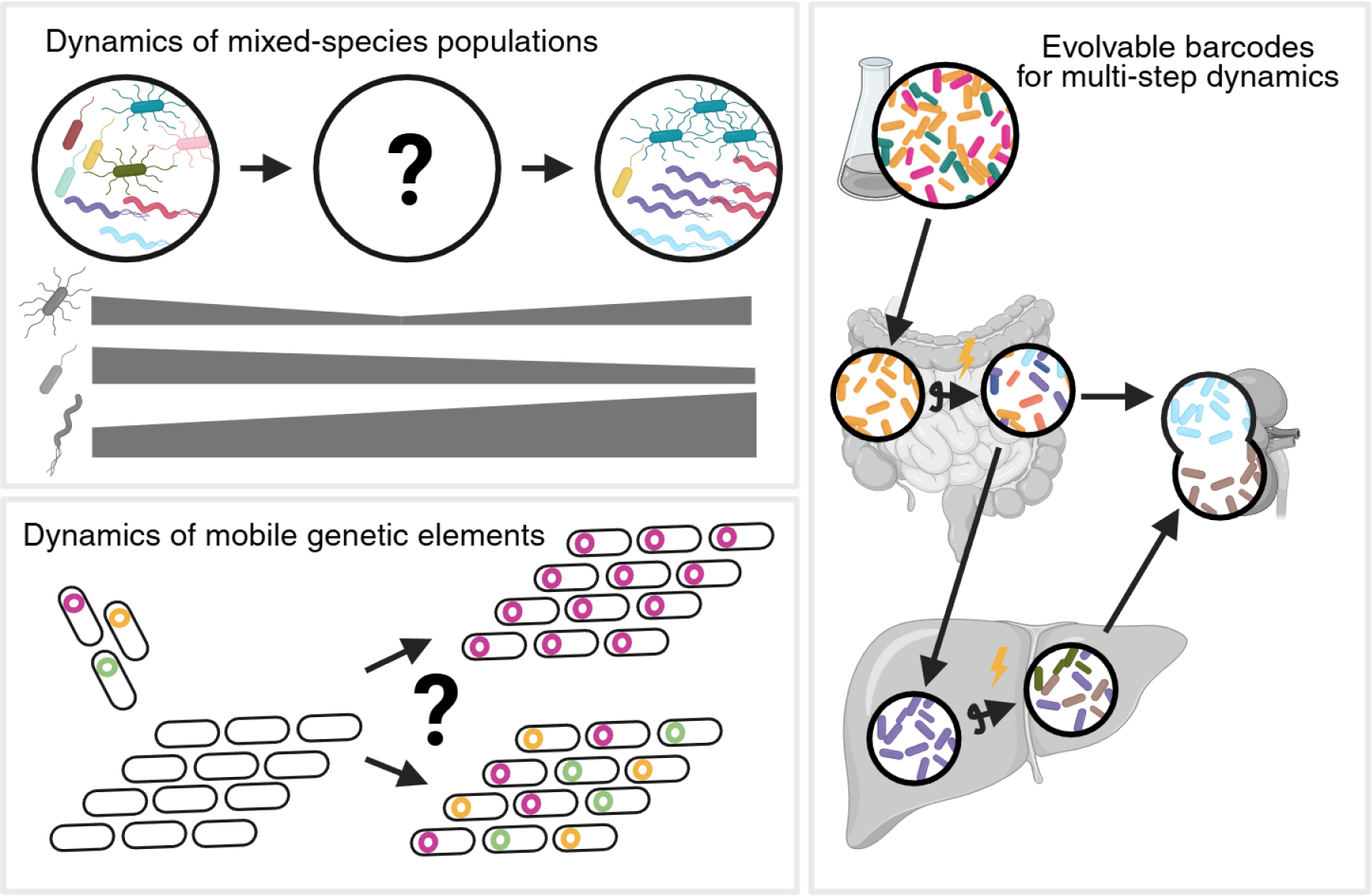
Future applications of barcoding to reveal microbial dynamics within the host. Introducing a mixed-species population into a host (e.g., during development or reconstitution of a germ-free animal) triggers a complex pattern of microbial succession that could be revealed using barcodes to map individual lineages. Barcodes could also be used to track the transfer of mobile genetic elements (e.g., plasmids) within the host. Furthermore, evolvable barcodes could reintroduce diversity to bottlenecked populations, deconvolving otherwise hidden patterns of migration. Created in BioRender.

One emerging use of barcoding is the simultaneous measurement of the relative fitness of multiple bacterial genetic mutants within a single host (58–61). An exciting future application is to leverage barcoding to measure dynamics among mixed-species populations, such as organisms in the intestinal microbiota or in polymicrobial infections (Fig. 5). For example, individual constituents of polymicrobial communities can be distinguished by different barcodes, facilitating rapid measurements of relative abundances in studies of ecological competition or cooperation. We also expect that the increased feasibility of barcoding will further expand its application to other microbial infection models, such as viruses and protozoa (62–67).

Barcoding approaches can reduce the number of participants and increase the information gained from challenge studies, which is particularly valuable in large-animal or controlled human models of infection (28, 68, 69). Previously, determining pathogen infectivity was accomplished by challenging multiple participants with increasing inocula. Using barcodes, these dosing studies can be downsized because lineage tracing provides information about the infectious bottleneck at every dose. Furthermore, we anticipate that application of barcoded bacteria to controlled human challenge studies will elucidate the mechanisms of vaccine protection, as well as the environmental (e.g., diet) and host (e.g., age and comorbidities) factors that control colonization bottlenecks and within-host microbial evolution.

Bacterial lineage tracing also offers a new perspective to grapple with critical public health concerns, including the increasing threat of antibiotic resistance (70). Combining barcoding with experimental bacterial evolution offers a powerful approach to define the trajectories of antimicrobial resistance (71–73). For example, exposure of a barcoded population to an antibiotic can reveal precisely how many organisms acquired resistance, and subsequent experiments can identify causal mutations and illuminate the impacts on infection outcome. Furthermore, antibiotic resistance often spreads through horizontal gene transfer, and we anticipate that the application of lineage tracing to track mobile elements will reveal the bottlenecks and within-host dynamics that govern the transfer of antimicrobial resistance between microbes (Fig. 5). Moreover, barcoding mobile genetic elements will uncover previously unappreciated processes that govern horizontal gene transfer.

Inspired by lineage tracing in eukaryotes, where technological advances have facilitated studies ranging from cancer progression to yeast evolution (74–82), similar advances will expand the utility of bacterial barcoding. Evolvable barcodes, which can introduce new diversity (Fig. 5), will facilitate the observation of population dynamics that occur after a diversity-limiting step, like a severe bottleneck or the sweep of a dominant mutant. New technologies may also reveal the mechanisms that create the founding population. Visualizable barcodes could illuminate the location of the founding population, elucidating the mechanisms that result in the replicative niche that sustains the founding population. Furthermore, a growing body of literature has underscored the significance of genotype-independent microbial heterogeneity within clonal populations (21–23), suggesting that the founding population could consist largely of the cells in the inoculum with the highest expression of important colonization factors, which could be tested by combining barcoding with single-cell RNA sequencing. Ultimately, synthesizing observations from diverse microbial pathogens and models will yield insight into universal quantitative principles of infectious diseases.

## Box 1. Bottlenecks, genetic drift, and ecology

Host-microbe bottlenecks have been analogized with the idea of genetic drift (20). For example, a fire may dramatically reduce the number of organisms in a forest, resulting in a change in the distribution of the residents’ genotypes. In this analogy, the fire creates a bottleneck, and the survivors are the founding population. While it is possible for certain genotypes to have fitness advantages (e.g., heat tolerance), the fire’s impact is largely independent of genotype because of its rapidity and severity, leading to genotype-independent outcomes. The short time scales and severity of infection bottlenecks create parallels with genetic drift because both result in genotype-independent, stochastic loss of diversity. However, the parallels to genetic drift are imperfect. Experimentally inoculated microbes generally lack the degree of genetic diversity present in natural contexts. Thus, any host-imposed bottlenecks are acting independently of bacterial genotype. Also, bacterial evolution can occur more rapidly than for diploid organisms, resulting in the emergence of dominant genotypes.

Lineage tracing frames studies of microbial populations within a broad ecological perspective. One notable example is priority effects, which refers to the impact of the first organisms that arrive in an ecosystem on later arrivals (21). In models of *Salmonella* and *L. monocytogenes* infection in mice, barcoding has been used to demonstrate that the first clones that arrive at the niche are more abundant than later arriving clones, a phenomenon also referred to as colonization resistance (22–24). Other ecological concepts, such as succession, community assembly, and interspecies competition also have parallels in host-microbe contexts and are quantifiable by lineage tracing.
